# Duck enteritis virus pUL47, as a late structural protein localized in the nucleus, mainly depends on residues 40 to 50 and 768 to 777 and inhibits IFN-β signalling by interacting with STAT1

**DOI:** 10.1186/s13567-020-00859-w

**Published:** 2020-11-11

**Authors:** Tianqiong He, Mingshu Wang, Anchun Cheng, Qiao Yang, Renyong Jia, Ying Wu, Juan Huang, Shun Chen, Xin-Xin Zhao, Mafeng Liu, Dekang Zhu, Shaqiu Zhang, Xuming Ou, Sai Mao, Qun Gao, Di Sun, XinJian Wen, Bin Tian, Yunya Liu, Yanling Yu, Ling Zhang, Leichang Pan, Xiaoyue Chen

**Affiliations:** 1 Institute of Preventive Veterinary Medicine, Sichuan Agricultural University, Wenjiang, Chengdu, 611130 Sichuan People’s Republic of China; 2 Key Laboratory of Animal Disease and Human Health of Sichuan Province, Sichuan Agricultural University, Wenjiang, Chengdu, 611130 Sichuan People’s Republic of China; 3 Avian Disease Research Center, College of Veterinary Medicine, Sichuan Agricultural University, Wenjiang, Chengdu, 611130 Sichuan People’s Republic of China

**Keywords:** DEV, UL47, NLS, localization, IFN-β, STAT1

## Abstract

Duck enteritis virus (DEV) is a member of the *Alphaherpesvirinae* subfamily. The characteristics of some DEV genes have been reported. However, information regarding the DEV UL47 gene is limited. In this study, we identified the DEV UL47 gene encoding a late structural protein located in the nucleus of infected cells. We further found that two domains of DEV pUL47, amino acids (aa) 40 to 50 and 768 to 777, could function as nuclear localization sequence (NLS) to guide the nuclear localization of pUL47 and nuclear translocation of heterologous proteins, including enhanced green fluorescent protein (EGFP) and beta-galactosidase (β-Gal). Moreover, pUL47 significantly inhibited polyriboinosinic:polyribocytidylic acid [poly(I:C)]-induced interferon beta (IFN-β) production and downregulated interferon-stimulated gene (ISG) expression, such as Mx and oligoadenylate synthetase-like (OASL), by interacting with signal transducer and activator of transcription-1 (STAT1).

## Introduction

Duck virus enteritis (DVE), is an acute, septic, and febrile disease that occurs in waterfowl of all ages, with a high mortality rate and a declined duck egg production, resulting in severe economic losses to duck industry [1]. A variety of vaccines have been developed to control this disease [2]. DVE is caused by duck enteritis virus (DEV), which is a member of the *Alphaherpesvirinae* subfamily; the genome is a linear double-stranded DNA molecule (approximately 150 kb in length) divided into a unique long (UL) region and a unique short (US) region flanked by an internal short repeat (IRS) and a short terminal repeat (TRS) [3].

Several studies have been reported regarding the UL47 gene. The herpes simplex virus-1(HSV-1) UL47 gene encodes two major tegument proteins, VP13 and VP14, which represent differently processed forms of post-translational modification (phosphorylation, glycosylation or nucleotidylylation); VP13/14 is an RNA binding protein and shuttles between the nucleus and cytoplasm [4], modulates the trans-acting activity of trans-inducing factor VP16 and stimulates viral immediate-early gene transcription in newly infected cells [5]. In addition, VP13/14 can elicit T cell responses, and three epitopes of VP13/14, including the residues from 286 to 294, 504 to 512, and 544 to 552, were recognized by multifunctional CD8^+^ T cells [6, 7]. VP8, the homologue of VP13/14 in bovine herpesvirus 1 (BoHV-1), which is also phosphorylated and glycosylated, shuttles between the nucleus and cytoplasm and binds the mRNA of gB, gC and ICP0 [8–10]. Furthermore, VP8 interacts with STAT1, heat shock protein-60 (HSP60), and DNA damage response proteins to regulate IFN-β production, apoptosis, and mitochondrial functions, respectively [11–13]. Nevertheless, the infectious laryngotracheitis virus (ILTV) UL47 gene exhibits several differences from those of other alphaherpesviruses; the conserved UL47 gene is translocated from the UL to the US region and located between the conserved US3 and US4 genes [14]. The UL47 genes of HSV-1 [15], ILTV [14], pseudorabies virus (PRV) [16, 17], BoHV-1 [18], varicella-zoster virus (VZV) [19] and Marek’s disease virus (MDV-1) [20] have also been demonstrated to be dispensable for viral replication in cell culture.

The properties of some DEV genes have been preliminary characterized [21–36]. We previously reported that DEV UL47-encoded protein (pUL47) modulates the distribution of UL41-encoded protein (pUL41) [37]. Together with the findings of previous studies [38, 39], we speculated that a close connection exists between pUL41 and pUL47, but the role of DEV pUL47 has not yet been defined. In this report, we explored the kinetics and location of pUL47, and we found DEV UL47 was a late gene encoding an abundant protein assembled in virion, and pUL47 was mainly localized to the nucleus in newly infected cells. In addition, residues 40 to 50 and 768 to 777 were necessary for nuclear localization of pUL47 and nuclear translocation of heterologous proteins. Moreover, we also explored and found pUL47 could interact with STAT1 to significantly inhibit IFN-β production and ISG expression.

## Materials and methods

### Viruses, cells and vectors

Duck embryo fibroblast (DEF) cells were cultured in minimal essential medium (MEM; Gibco, Meridian Road Rockford, USA) supplemented with 10% (v/v) foetal bovine serum (FBS; Gibco, Meridian Road Rockford, USA) at 37 °C with 5% CO_2_. In this study, the DEV CHv strain (GenBank: JQ647509.1) was isolated [40] and propagated on DEF cells with 2% FBS MEM. Virus-containing media was collected and centrifuged to remove large cellular debris, and then stored at − 80 °C until use.

The pcDNA3.1(+)-UL47, pcDNA3.1(+)-UL47Δ40-50, pcDNA3.1(+)-UL47Δ768-777, pEGFP-C2-SV40, pEGFP-C2-β-gal, pEGFP-C2-SV40-β-gal plasmid, and IFN-β-Luc reporter plasmid expressing firefly luciferase under the control of the duck IFN-β promoter were prepared in our laboratory [41].

### Construction of the recombinant expression vector

All primers were designed by Primer Premier 5 software (Table 1). The wild-type DEV UL47 (GenBank: AFC61841.1) major antigenic domains (1417–2353 bp) were cloned into pMD18T (Novagen, Podenzano, Italy) and then sub-cloned into pET-32a (+) vector (Novagen, Podenzano, Italy) to create pET-32a (+)-UL47. We further constructed the eukaryotic plasmid pcDNA3.1(+)-Flag-UL47, and then deleted the 40 to 50 aa (RRSGKRRTLDR) and 768 to 777 aa (KALKRRLTGG) of pUL47, named pcDNA3.1(+)-Flag-UL47Δ40-50-768-777. Moreover, we fused residues 40 to 50 and 768 to 777 of DEV pUL47 to pEGFP-C2 or pEGFP-C2-β-gal, named pEGFP-C2-40-50, pEGFP-C2-768-777, pEGFP-C2-40-50-β-gal and pEGFP-C2-768-777-β-gal, respectively. In addition, the entire UL47 open reading frame (ORF) and duck STAT1 (Gene ID: 101795022) ORF were inserted into the pcaggs vector, named pcaggs-UL47-Flag and pcaggs-STAT1-myc, respectively. UL47 ORF with an LgBiT tag and STAT1 ORF with an SmBiT tag were inserted into the C- or N-termini of pcDNA3.1(+) vector, named pcDNA3.1-C-UL47-LgBiT and pcDNA3.1-N-STAT1-SmBiT, respectively.

**Table 1:** Sequences and primer pair characteristics

Primer	Plasmid	Primer sequence (5′ → 3′)
P_1_	pET-32a-UL47	G/GATCCAGACAGCGCCGTAGGTCA
P_2_		C/TCGAGAACTATTGCCGGATTAACAGG
P_3_	DEV UL47 (RT-PCR)	AACGGAGTTGCTTGGAGAACA
P_4_		TGGGCGATGAAACAGAGTAGG
P_5_	Duck β-actin (RT-PCR)	CCGGGCATCGCTGACA
P_6_		GGATTCATCATACTCCTGCTTGCT
P_7_	DEV UL54 (RT-PCR)	GAACAACCGCCGAACAC
P_8_		TCAAACATCCGCCTCAA
P_9_	DEV UL13 (RT-PCR)	GCCACCAACCCTACCAAG
P_10_		GTCGTCAGCCCATCACCA
P_11_	DEV Us2 (RT-PCR)	AGACGGTTCCGAAAGTACAG
P_12_		TCGGCAGCACCAATAATCC
P_13_	pcaggs-UL47-Flag	CATCATTTTGGCAAAG/AATTCGCCACCATGGCCATGGATAAATCACGAAGAC
P_14_		TTGGCAGAGGGAAAAA/GATCTTTACTTGTCATCGTCGTCCTTGTAATCATGTAACTCTCTCCGCCC
P_15_	pcasggs-STAT1-myc	CATCATTTTGGCAAAG/AATTCGCCACCATGGCCGAGCAGAAACTCATCTCTGAAGAGGATCTGATGACTCAGTGGTACCAG
P_16_		GGCAGAGGGAAAAA/GATCTTTAAGTTGAATATGCTGAACAC
P_17_	pEGFP-C2-40-50	AATTCAGGAGAAGTGGTAAGAGACGTACACTTGACAGGG
P_18_		GATCCCCTGTCAAGTGTACGTCTCTTACCACTTCTCCTG
P_19_	pEGFP-C2-768-777	AATTCAAAGCATTAAAACGACGTTTGACTGGTGGGG
P_20_		GATCCCCCACCAGTCAAACGTCGTTTTAATGCTTTG
P_21_	pEGFP-C2-40-50-β-gal	CTCGAGCTCAAGCTTCG/AATTCAGGAGAAGTGGTAAGAGACGTACACTTGACAGGATGAGCGAAAAATACATCG
P_22_		CAGTTATCTAGATCCGGTG/GATCCTTATTTTTGACACCAGAC
P_23_	pEGFP-C2-768-777-β-gal	CTCGAGCTCAAGCTTCG/AATTCAAAGCATTAAAACGACGTTTGACTGGTGGGATGAGCGAAAAATACATCG
P_24_		CAGTTATCTAGATCCGGTG/GATCCTTATTTTTGACACCAGAC
P_25_	pcDNA3.1 (+)-Flag-UL47Δ40-50-768-777	GCTTGGTACCGAGCTCG/GATCCGCCACCATGGATTACAAGGATGACGACGATAAGATGGATAAATCACGAA
P_26_		GTTTAAACGGGCCCTCTAGAC/TCGAGTTAATGTAACTCTCTCCGC
P_27_	pcDNA3.1-C-UL47-LgBiT	CTGTTGGTAAAGCCACCA/GATCTGCCACCATGGATAAATCACGAAG
P_28_		CCGCTCCCGCCACCACCGC/TCGAGGGCTTGTCATCGTCGTCCTTGTAATCATGTAACTCTCTCCGCCC
P_29_	pcDNA3.1-N-STAT1-SmBiT	GAGCGGAGGTGGAGGC/TCGAGCATGACTCAGTGGTACCAG
P_30_		CCGCCCCGACTCTAGAA/GATCTTTACAGATCCTCTTCAGAGATGAGTTTCTGCTCAGTTGAATATGCTGAACAC

### Preparation of the polyclonal antibody

pET-32a(+)-UL47 was transformed into *E. coli* BL21 (DE3). Freshly transformed bacteria were inoculated into LB medium (100 µg/mL Amp) at 37 °C until the absorbance at 600 nm reached 0.4–0.6 and then expressed under the IPTG (0.2 mM) induction for 6 h at 37 °C. The vector control culture was analysed in parallel. The bacteria were collected by centrifugation (10 000 × *g*, 15 min, 4 °C) and disrupted by ultrasonication. The fusion protein was purified by gel and immunized in rabbit with Freund’s adjuvant to prepare a specific polyclonal antibody.

### SDS-PAGE and western blotting analysis

Samples containing 20 µL of total proteins were boiled for 10 min; 5 µL of 5x SDS loading sample buffer was added, and the samples were centrifuged (10 000 × *g*, 5 min, 4 °C). Then, 10 µL of supernatant of each sample was analysed by sodium dodecyl sulphate polyacrylamide gel electrophoresis (SDS-PAGE). The samples were transferred to polyvinylidene fluoride (PVDF) membranes. The membranes were blocked for 2 h in 5% skim milk at 37 °C and then incubated with primary antibody and probed with HRP-conjugated secondary antibody (Bio-Rad Lab, CA, USA) for 1 h at 37 °C, respectively [43]. All antibodies were diluted in 1% skim milk. After several rinses with TBST (containing 0.1% Tween-20) to remove unbound antibodies, proteins were detected with Western BLoT Chemiluminescence HRP Substrate (Takara, Dalian, China) according to the manufacturer’s instructions. The following antibody were used: rabbit anti-DEV antibody (1:800), rabbit anti-UL47 polyclonal antibody (1:1000), mouse anti-actin antibody (1:5000, Proteintech-66009-1), mouse anti-GFP antibody (1:1000, Beyotime-AG281-1), mouse anti-Flag MAb (1:5000, MBL-M185-3) and mouse anti-Myc MAb (1:10 000, MBL-M192-3).

### Indirect immunofluorescence assay (IFA)

DEV-infected DEF cells in 6-well dishes were collected on glass coverslips at 0, 9, 20, 36, 48, 60, and 72 h post-infection (hpi), fixed with 4% paraformaldehyde for 30 min, and permeabilized with 0.25% Triton X-100 for 30 min at 4 °C. The cells were rinsed three times with PBST (containing 0.1% Tween-20) for 5 min each time and blocked for 2 h with 5% BSA PBS at 37 °C. Then, the cells were incubated with primary antibodies and secondary antibodies. All antibodies were diluted in 1% BSA PBS. Finally, the cell nuclei were visualized with DAPI (Roche, Mannheim, Germany) for 15 min at room temperature. Coverslips were sealed with glycerine buffer and kept in a humidified chamber to avoid evaporation. The coverslips were visualized using a fluorescence microscope (Nikon ECLIPSE 80i, Japan).

### RT-qPCR

Total RNA was collected using TRIzol reagent l (Invitrogen, CA, USA) according to the manufacturer’s recommendations, and complementary DNA (cDNA) was generated using the PrimeScript^®^RT reagent kit with the gDNAeraser (Takara, Beijing, China). Target genes were detected using the previously described primers (Table 1), and β-actin was used as internal reference as previously described [21]. All reactions were performed in triplicate and in at least three independent experiments. The relative levels of gene expression were determined with the 2^−ΔΔ*C*t^ method.

### Luciferase reporter assays

DEF cells were co-transfected with 400 ng/well of pcaggs-UL47-Flag or an empty vector, 400 ng/well of reporter plasmid IFN-β-Luc, and 4 ng/well of pRL-TK (Promega, USA) using Lipofectamine 3000, and the cells were stimulated with 50 ng/ml poly(I:C) at 12 h after transfection and harvested at 24 h after stimulation. Finally, we detected the firefly luciferase activity by the dual-luciferase assay system (Promega, USA) according to the manufacturer’s recommendations. All reactions were performed in triplicate and at least three independent experiments.

### NanoLuc Binary Technology (NanoBiT) assay [44]

BHK21 cells in growth medium were seeded in white 96-well plates (Costar 3917) at 37 °C and 5% CO_2_. The cells were co-transfected with pcDNA3.1-C-UL47-LgBiT and pcDNA3.1-N-STAT1-SmBiT using Lipofectamine 3000 according to the instructions; FKBP-SmBiT and FRB-LgBiT were used as positive controls, whereas Halotag-SmBiT coexpressed with pcDNA3.1-C-UL47-LgBiT, pcDNA3.1-C-UL47-LgBiT coexpressed with FKBP-SmBiT and pcDNA3.1-N-STAT1-SmBiT coexpressed with FRB-LgBiT were used as negative controls. The medium was replaced with serum-free media, and luciferase was detected after 20 h posttransfection. Luminescence was measured after addition of 25 μl Nano-Glo Live cell reagent (Promega, N2012) prepared according to the manufacturer’s protocol. All reactions were performed in triplicate and in at least three independent experiments.

### Virion purification

DEV CHv strain was propagated in 9-day-old duck embryos. The embryos that died at 36–72 hpi were harvested. The allantoic fluids from the duck embryos were collected to purify the virion. In brief, clarified supernatants were underlaid with 5 mL 30% (w/v) sucrose in PBS and centrifuged in a Beckman Ti70 rotor (40 000 × *g*, 2 h, 4 °C). The pellet was finally diluted in PBS and stored at − 80 °C.

### Mass spectrometry

Purified virion samples were boiled for 10 min with 5x SDS loading sample buffer and then analyzed by 12% SDS-PAGE. The whole gel was stained with Coomassie brilliant blue (Sigma) and then sent to Sangon Biotech Company (Shanghai, China) for liquid chromatography-tandem mass spectrometry (LC–MS/MS) analysis.

### DEV UL47 expression kinetics

During the productive cycle, herpesviruses strictly exhibit temporal cascades of gene expression that are divided into three stages: immediate early (IE), early (E), and late (L); L genes strictly requires the onset or completion of lytic DNA amplification [36]. To identify the DEV UL47 expression kinetics, DEF cells were infected with DEV and incubated at 37 °C for 2 h with 300 µg/mL DNA polymerase inhibitor, ganciclovir (GCV) or 100 µg/mL protein synthesis inhibitor, cycloheximide (CHX), and the medium was replaced with 2% FBS MEM including the drugs. Total RNA was extracted at 24 hpi with TRIzol and subsequently reverse transcribed into cDNA. PCR was conducted using primers confirmed on a 1% agarose gel.

### Statistical analysis

The comparisons between the different groups were made by one-way ANOVA with GraphPad Prism 7.0 software (La Jolla, CA, USA). All experiments were repeated at least three times individually. The data was expressed as the mean and standard error of the mean (SEM). Values of *p < 0.05 were considered statistically significant.

## Results

### Preparation of a specific rabbit anti-UL47 polyclonal antibody

**Figure 1:**
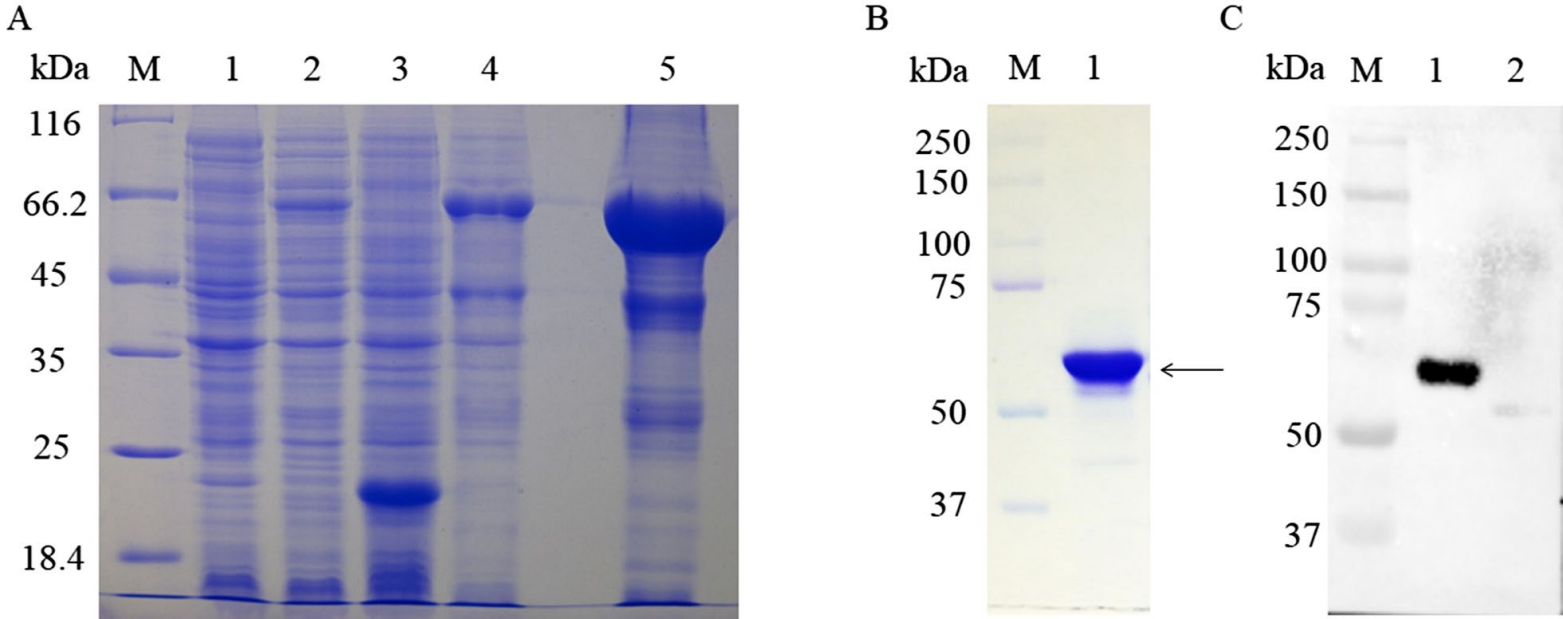
**Purified fusion protein and western blot analysis. A** Lane 1, pET32a-UL47, non-induced; Lane 2, pET-32a-UL47 recombinant bacteria after induction in *E. coli* BL21 (DE3); Lane 3, pET-32a(+) vector; Lane 4, pET-32a-UL47 recombinant bacterial supernatant; Lane 5, pET-32a-UL47 recombinant bacterial sediment. **B** Purification of the recombinant protein. Lane 1, purified protein (approximately 55 kDa); **C** Expressed protein was recognized with rabbit anti-DEV antibody (1:800). Lane 1, recombinant protein (approximately 55 kDa); Lane 2, pET-32a (+) vector; M. Precision Plus Protein™ Dual Color Standards.

**Figure 2:**
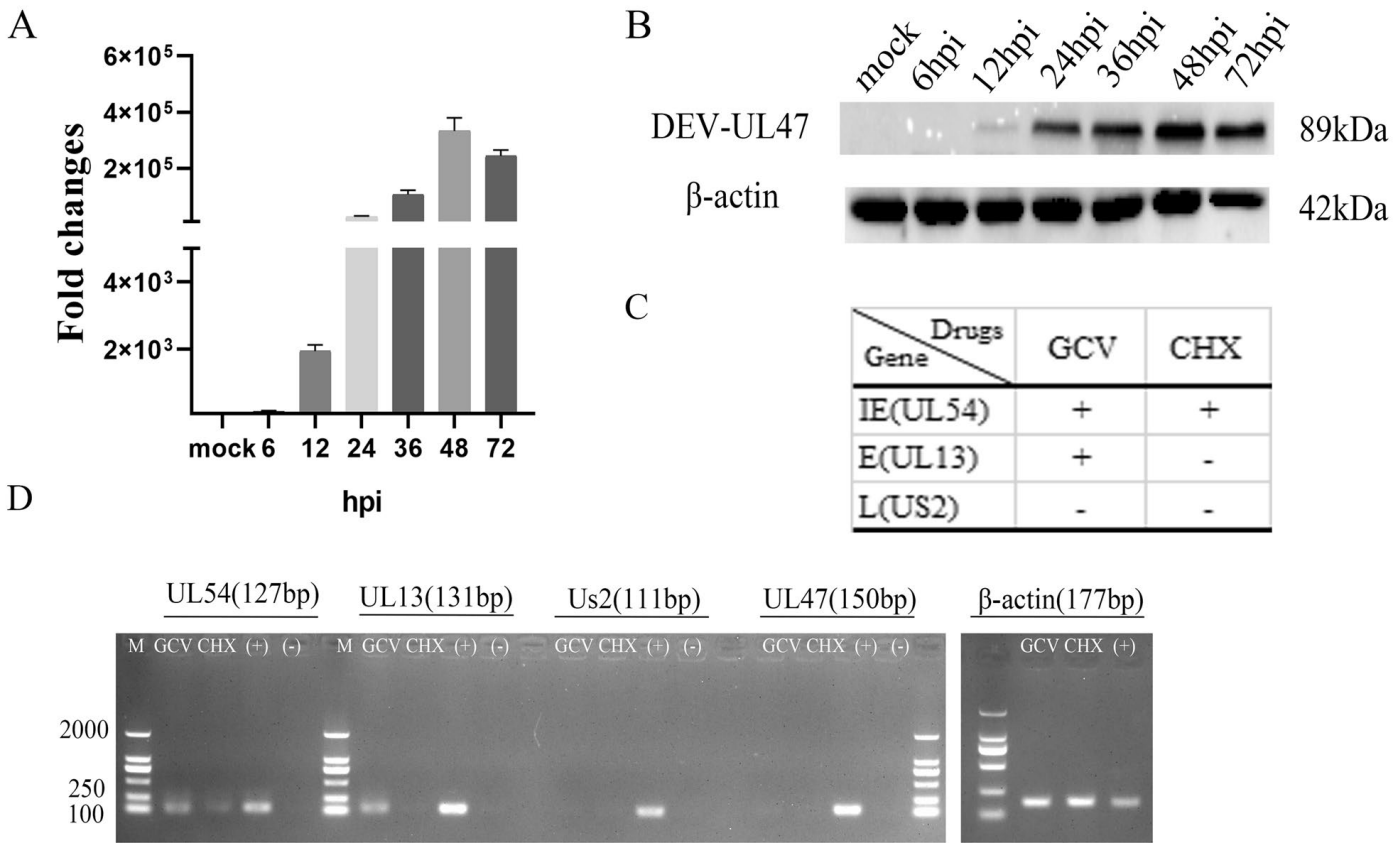
**The DEV UL47 gene is a late gene. A** Total RNA isolated from mock-infected and DEV-infected cells at various times was analysed using RT-qPCR and normalized to β-actin, and plotted the fold change over mock. **B** The expression analysis of the UL47 gene product in DEV-infected DEFs at different time points. The samples derived from the same experiment and gels were processed in parallel. **C** Schematic diagram showed the IE gene (UL54), E gene (UL13), and L gene (US2). **D** GCV group: DEV-infected cells treated with GCV; CHX group: DEV-infected cells treated with CHX. (+) group: DEV-infected cells without drugs. M, DL2000 Marker.

The expressed product was a UL47-his fusion protein (approximately 55 kDa) that was largely present in insoluble form but not in the vector control culture (Figure 1A). Next, the fusion protein was purified by gel and electric elusion (Figure 1B), detected with the rabbit anti-DEV antibody by western blotting [37] (Figure 1C), and then immunized in rabbit with Freund’s adjuvant to prepare a specific anti-UL47 polyclonal antibody. The pUL47 in the DEV-infected cells reacted strongly with this specific polyclonal antibody (Figure 2B).

### The DEV UL47 gene is a late gene

As shown in Figure 2A, the UL47 gene transcript level increased gradually from 6 hpi to 48 hpi and peaked at 48 hpi followed by a steady decrease.

We further identified the expression of the DEV UL47 gene. In lysates of DEV-infected cells, the UL47-specific antiserum recognized an 89 kDa major protein (Figure 2B). The band was not detected in the mock group and 6hpi and increased in amount until 48 hpi, with a steady decrease at 72 hpi. The β-actin antibody was used to assess β-actin as the loading control.

In addition, we treated the infected cells with GCV or CHX, and then collected them at 24 hpi to detect viral genes. The correct bands of β-actin (177 bp) and the IE gene UL54 (127 bp) [42], E gene UL13 (131 bp) [45], and L gene Us2 (111 bp) [36] were shown (Figure 2C), and the UL47 band (150 bp) was detected in the positive group but not in the GCV, CHX or negative groups (Figure 2D). These results showed that DEV UL47 gene was L gene due to its strict dependence on the early synthesis of viral DNA and protein.

### pUL47 is a component of the virion

**Figure 3:**
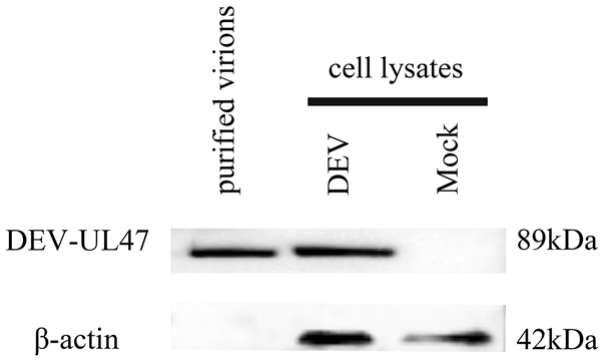
**DEV pUL47 is a component of the virion.** The purified DEV virions were identified using rabbit anti-UL47 antibody serum. As a control, no band was present in the DEF cell group.

Mass spectrometry was used to identify the protein content of extracellular virions. The results showed that pUL47 was present in mature extracellular virions. Based on the exponentially modified protein abundance index (emPAI), the relative abundance of UL47 was far higher than that of gC, seventeen unique DEV UL47 peptides were detected, while three unique peptides matched the DEV gC (P < 0.05) (Table 2). Furthermore, the DEV pUL47 band was detected in virus-infected cells and virion particles (Figure 3A).

**Table 2:** Viral content of DEV extracellular virions (partial)

Protein	Information	Score	Mass	Matches	Sequences	emPAI	NCBI accession
UL44	Glycoprotein C	97	47 836	6 (3)	6 (3)	0.22	AJG04885
UL47	Tegument protein	580	88 548	27 (17)	18 (11)	0.72	AJG04881

### DEV UL47 protein mainly localizes to the nucleus in newly infected cells

**Figure 4:**
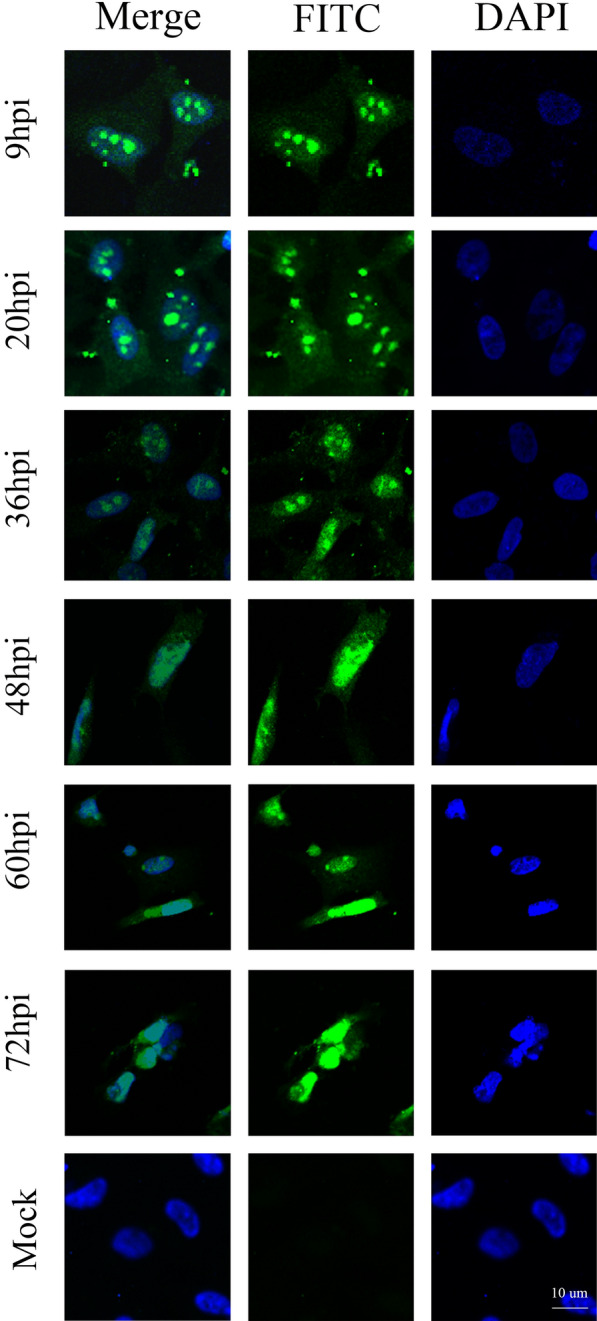
**pUL47 mainly localizes in the nucleus of DEV-infected cells.** Representative DEV pUL47 localization (green in all images). The mock group included untreated DEF cells using rabbit anti-UL47 polyclonal antibody and goat anti-rabbit IgG (H + L) cross-adsorbed secondary antibody, Alexa Fluor 568 (Invitrogen, 1:1000) (magnification: 200×; scale bar: 10 μm).

To analyse the intracellular localization of the pUL47, IFA was performed on DEF cells infected with DEV. The results showed that pUL47-specific fluorescence (green) was mainly located in the nucleus as well as diffused in the cytoplasm, and the signal became gradually stronger over time (Figure 4). In some nuclei, the protein accumulated in the nucleoli (9–36 hpi) and appeared diffuse (48–72 hpi). No fluorescence was observed in the mock-infected cells.

### Two small original motifs of UL47 function as an NLS

**Figure 5:**
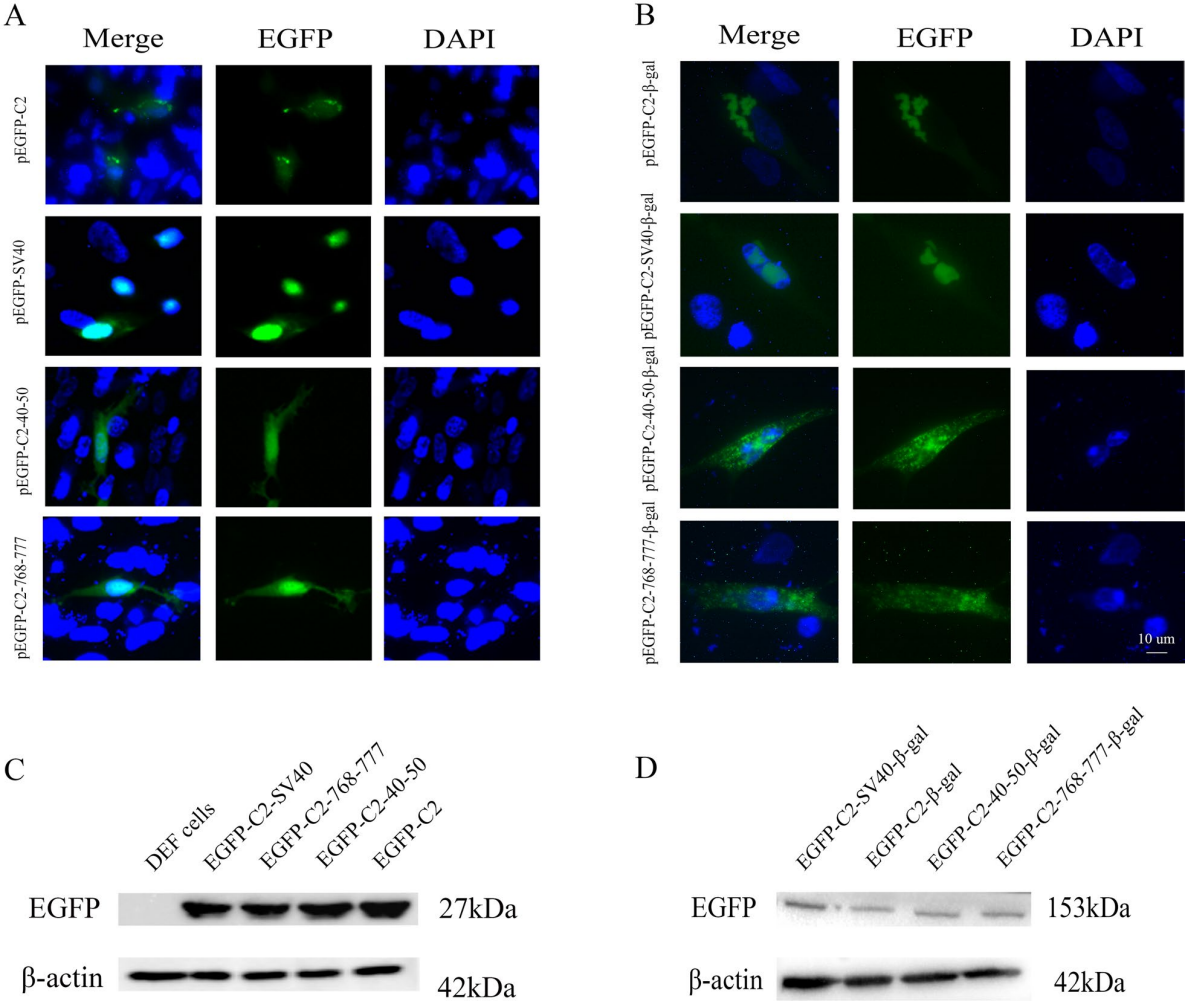
**The residues 40–50 and 768–777 of DEV pUL47 could direct heterologous proteins to the nucleus.** All transfected samples were collected after 24 h posttransfection. **A** DEF cells were transfected with pEGFP-C2-SV40, pEGFP-C2-40-50, pEGFP-C2-768-777 and pEGFP-C2, respectively. **B** The expression of proteins was confirmed by Western blotting using mouse anti-GFP monoclonal antibody and goat anti-mouse IgG HRP-conjugated antibody (1:5000) (magnification: 200×; scale bar: 10 μm). **C** DEF cells were transfected with pEGFP-C2-SV40-β-gal, pEGFP-C2-40-50-β-gal, pEGFP-C2-768-777-β-gal and pEGFP-C2-β-gal, respectively. **D** The expression of proteins was confirmed by western blotting using mouse anti-GFP monoclonal antibody.

According to previous results [37], we further predicted the putative basic region of DEV pUL47 using software at https://www.predictprotein.org/. The corresponding sequences (residues 40 to 50 and 768 to 777 of pUL47) were fused to pEGFP-C2 and transfected into DEF cells. The fusion proteins EGFP-C2-40-50 and EGFP-C2-768-777 were mostly localized in the nucleus (Figure 5A). As controls, DEF cells were transfected with pEGFP-C2 or with a vector expressing EGFP carrying NLS of SV40 large T antigen, leading to cytoplasmic or nuclear localization, respectively.

Because of the small molecular weight of EGFP, which enables its passive diffusion, we further constructed the plasmids pEGFP-C2-40-50-β-gal and pEGFP-C2-768-777-β-gal, encoding a fusion protein containing a large-galactosidase cytoplasmic protein, avoiding the passive diffusion phenomenon. As shown in Figure 5B, the fusion proteins EGFP-C2-40-50-β-gal and EGFP-C2-768-777-β-gal were distributed in the nuclei of DEF cells. As controls, the DEF cells were transfected pEGFP-C2-β-gal and pEGFP-C2-SV40-β-gal showed an exclusively cytoplasmic and nuclear localization, respectively. The following results showed that these two small original motifs should harbour the main NLS.

### The amino acids 40 to 50 and 768 to 777 of DEV pUL47 are necessary for nuclear localization

**Figure 6:**
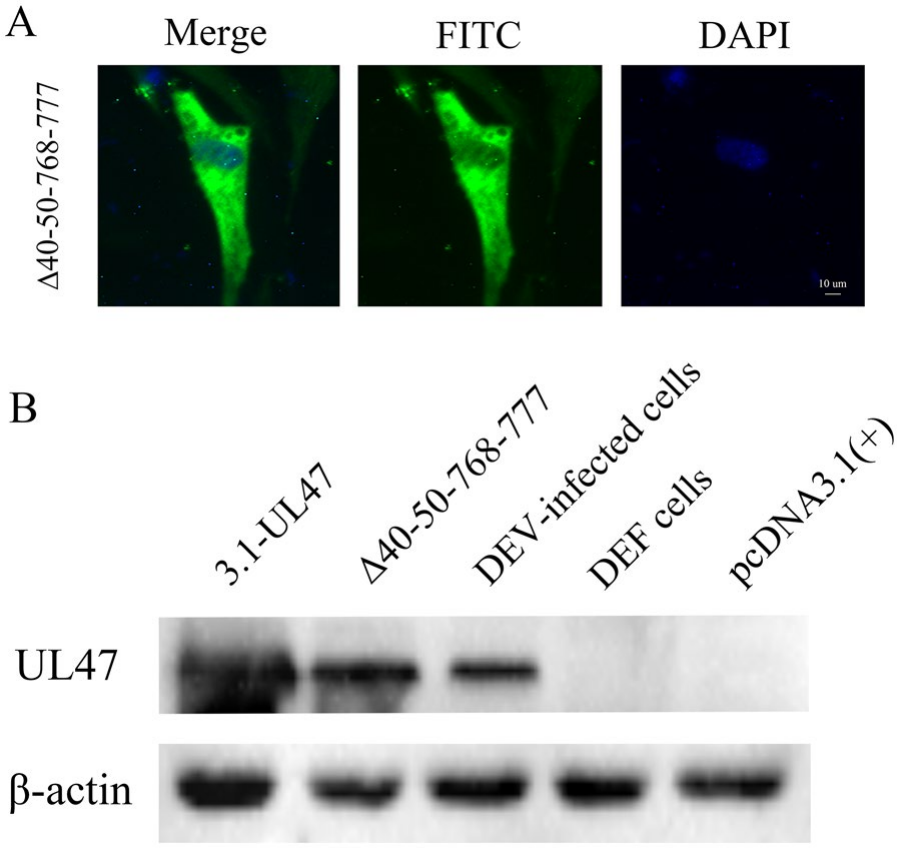
**The residues 40 to 50 and 768 to 777 of DEV pUL47 are necessary for nuclear localization. A** Distribution of the UL47 gene in DEF cells transfected with pcDNA3.1-Flag-UL47Δ40–50-768-777 (magnification: 200×; scale bar: 10 μm). **B** The expression of proteins was confirmed by western blotting using rabbit anti-UL47 polyclonal antibody.

According to the Ref. [37], we found that the residues 40 to 50 and 768 to 777 affected the nuclear localization of pUL47. In addition, we further identified that these two signals could translocate the EGFP and β-gal into the nucleus. When the residues 40 to 50 and 768 to 777 of pUL47 were deleted, the mutated protein (green) was mostly located in the cytoplasm, a small part of the pUL47 was still located in the nucleus (Figure 6A), which showed that DEV pUL47 might have another fragment to affect nuclear localization.

### DEV pUL47 inhibits IFN-β signalling by targeting STAT1

**Figure 7:**
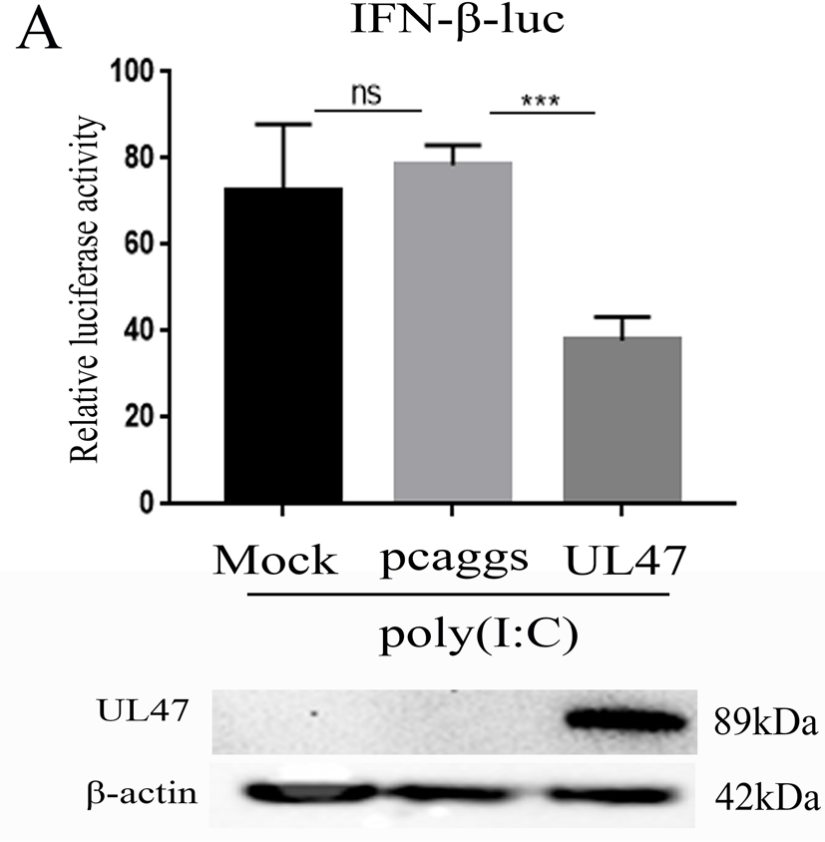
**DEV UL47 inhibits IFN-β signalling induced by poly(I:C).** All transfected samples were collected at 36 h posttransfection. **A** DEV UL47 inhibited the IFN-β-luc luciferase activation induced by poly(I:C) in DEF cells. DEF cells were co-transfected with IFN-β-luc luciferase reporter plasmid, pRL-TK, pcaggs-UL47-Flag or an empty vector, and then stimulated by 50 µg/mL at 12 h posttransfection. The cells were harvested and detected by dual-luciferase assay at 24 h posttransfection. Protein expression was confirmed by western blotting using mouse anti-Flag MAb. All experiments were performed in triplicate (n = 3). The data was analyzed by one-way ANOVA. ***p < 0.001

We further explored whether the pUL47 could inhibit IFN-β signalling. DEF cells were co-transfected with UL47-Flag plasmid or an empty vector and IFN-β-luc reporter plasmid, stimulated by the dsRNA analogues poly(I:C) stimulator, and then subjected to dual-luciferase reporter (DLR) assays. As shown in Figure 7A, DEV pUL47 significantly inhibited the IFN-β-luc activity induced by poly (I:C).

**Figure 8:**
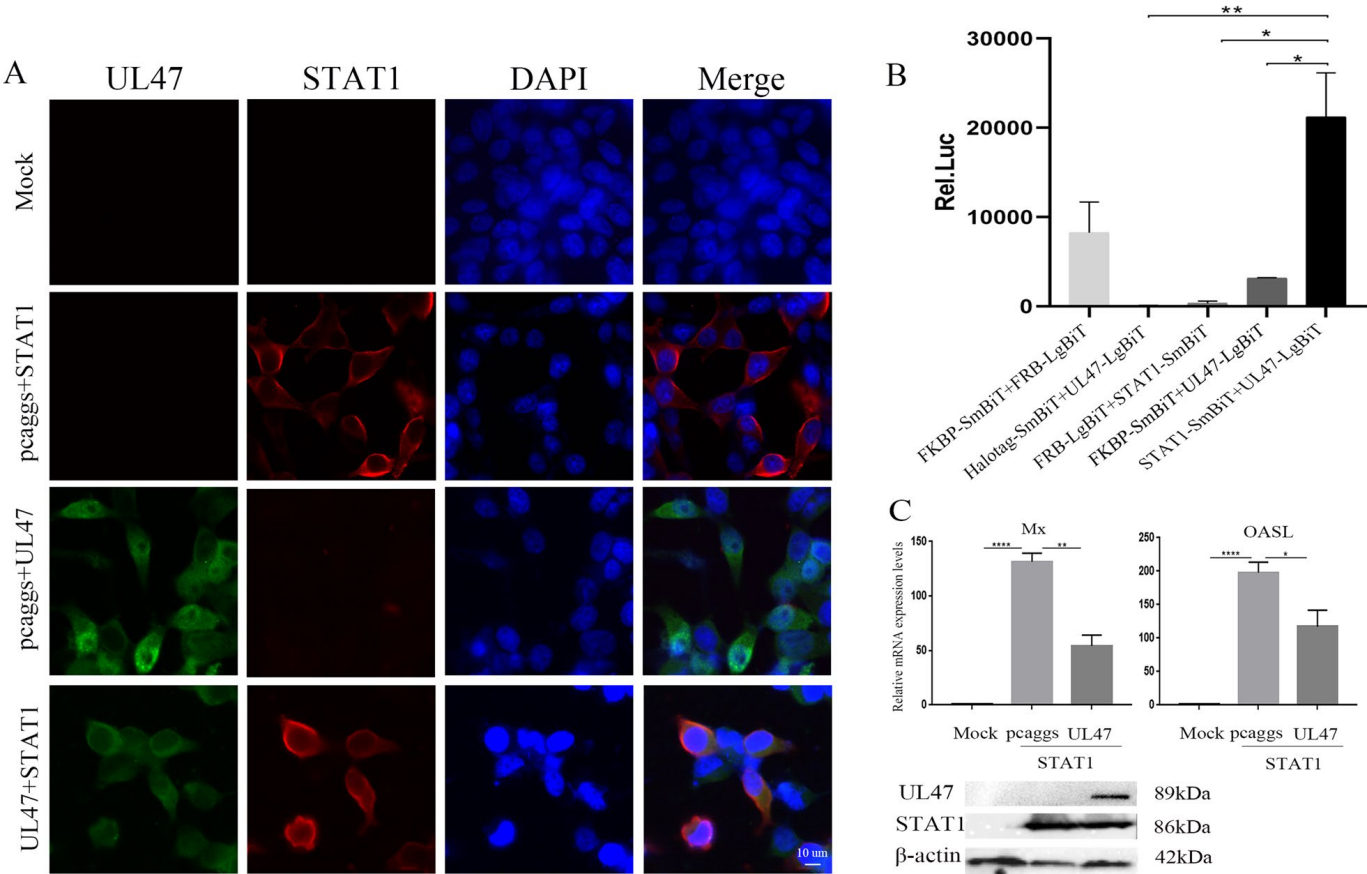
**pUL47 interacted with STAT1 in the cytoplasm and inhibited ISG expression induced by STAT1. A** HEK293T cells were coexpressed pcaggs-STAT1-myc with pcaggs-UL47-Flag, pcaggs-STAT1-myc with an empty vector and pcaggs-UL47-Flag with an empty vector (magnification: 200×; scale bar: 10 μm). **B** BHK21 cells contained pcDNA3.1-C-UL47-LgBiT coexpressed with pcDNA3.1-N-STAT1-SmBiT, FKBP-SmBiT coexpressed with FRB-LgBiT as positive controls, Halotag-SmBiT coexpressed with pcDNA3.1-C-UL47-LgBiT, pcDNA3.1-C-UL47-LgBiT coexpressed with FKBP-SmBiT and pcDNA3.1-N-STAT1-SmBiT coexpressed with FRB-LgBiT as negative controls. The luciferase signal was detected via NanoBiT after 20-h posttransfection. **C** DEV UL47 inhibited the mRNA levels of Mx and OASL induced by STAT1. DEF cells were co-transfected with pcaggs-STAT1-myc and pcaggs-UL47-Flag or an empty vector. The cells were harvested and detected by RT-qPCR at 36 h posttransfection. Protein expression was confirmed by Western blotting. All experiments were performed in triplicate (n = 3). The data was analyzed by one-way ANOVA. *P < 0.05, **P < 0.01 and ****P < 0.0001.

According to previous reports, BoHV-1 VP8 inhibited IFN-β signalling by interacting with and preventing the nuclear localization of STAT1 [11]. Therefore, we explored the relationship between DEV pUL47 and duck STAT1. DEV pUL47 co-localized with STAT1 in the cytoplasm (Figure 8A). Next, we detected the interaction between UL47 and STAT1, and the experimental group (co-transfected with pcDNA3.1-C-UL47-LgBiT and pcDNA3.1-N-STAT1-SmBiT) demonstrated a stronger signal than the positive group (co-transfected with FKBP-SmBiT and FRB-LgBiT); however, the negative control groups were not activated (Figure 8B). These results showed that DEV pUL47 interacted with STAT1 in the cytoplasm. Based on these results, we further explored whether pUL47 inhibited the STAT1-induced ISG expression. DEF cells were co-transfected with UL47-Flag or empty vector with STAT1-myc and then detected by RT-qPCR. As shown in Figure 8C, DEV pUL47 significantly reduced the expression of Mx and OASL proteins induced by STAT1. These results showed that DEV pUL47 targeted STAT1 to inhibit IFN-β signalling and host innate immunity.

## Discussion

Herpesviruses assemble more than 30 proteins into mature virions, which contain the nucleoprotein core, capsid shell, tegument and envelope. The tegument, which is a complex structure contained numerous viral gene products that play crucial roles in viral replication and the interaction of the virus with the host immune system [46, 47]. The UL47 gene is conserved in the *Alphaherpesvirinae* subfamily [48, 49], and pUL47 as a late abundant tegument protein in HSV-1 [50], BoHV-1 [51], MDV [52], ILTV [53] and PRV [54]. In addition, HSV-1 VP13/14 forms a complex with the viral proteins UL34, UL31, and/or US3, which regulate primary envelopment during viral nuclear egress [55]. Further research showed that VP13/14 interacts with the capsid protein pUL17, providing another link between the tegument and nucleocapsid [56]. PRV pUL47 plays a role in virion morphogenesis at the late stage of infection, and deletion of UL47 impairs the secondary envelopment, resulting in intracytoplasmic aggregation of tegumented capsids [16]. MDV pUL47 also regulates the expression of the UL46-UL49 locus [52]. However, BoHV-1 UL47 cannot affect the secondary envelopment [18]. In this study, we further found that the DEV UL47 gene encoded a late abundant structural protein assembled in the virion. Based on the high level of emPAI, the large abundance of this protein indicates an important function, requiring further exploration of the role of DEV pUL47 in the viral life cycle.

All the UL47 proteins encoded by HSV-1 [57], BoHV-1 [10], ILTV [53] and PRV [16] exhibit nuclear localization, suggesting an important role of these proteins in the nucleus. In addition, VP13/14 and VP8 can bind RNA and play a pivotal role in RNA transport. Our results have also shown that DEV pUL47 is mainly localized in the nucleus at early stage of infection and diffused in the cytoplasm at the late stage of infection. We speculated that DEV pUL47 could shuttle between the nucleus and cytoplasm. Whether DEV pUL47 has the same function to modulate the transcription remains to be elucidated.

Nuclear localization mainly depends on the nuclear targeting signal. The best-characterized nuclear targeting signal is the classical nuclear localization sequence (cNLS) [58, 59]. The cNLS are formed by one or two basic clusters of amino acid residues, termed monopartite or bipartite NLSs, respectively, and have the following consensus sequences: K(K/R)X(K/R) for monopartite cNLS and KRX_10-12_K(K/R)X(K/R) for bipartite cNLS (X corresponds to any residue) [60]. We predicted two putative basic regions of DEV pUL47 using software: monopartite NLS in the N-terminus (40RRSGKRRTLDR50) and C-terminus (768KALKRRLTGG777). In this report, we further identified that these sequences affect the UL47 distribution and direct EGFP and β-gal proteins into the nucleus. However, no cNLS region exists in VP13/14 or VP8; others have identified nonclassical arginine-rich nuclear localization signals at the N-terminus of VP13/14 and VP8, which were sufficient to direct a heterologous protein to the nucleus [4, 10], and the arginine-rich NLS of VP13/14 is associated with the RNA binding motif [61]. We also found a similar arginine-rich region in DEV pUL47 (125RRRR128); however, the prediction outcomes showed that the NLS score is negative. Based on the prediction outcomes, we deleted the residues 40 to 50 and 768 to 777 of DEV pUL47, a small part of pUL47 was still located in the nucleus (Figure 6A). Therefore, we speculated that the motif may also affect the nuclear localization.

In addition, VP13/14 forms a stable complex with viral kinase US3 and they reciprocally regulate subcellular localization in infected cells; US3 phosphorylation of VP13/14 (Ser-77) near its NLS (residues 50 to 68) appears to promote nuclear localization of VP13/14 in infected cells [62]. Furthermore, VP8 is also phosphorylated by US3, and the phosphorylation sites are near putative NLSs of VP8 [63]. The phosphorylation site of a protein near its NLS mediates its nuclear localization for a number of viral proteins [64]. Therefore, in addition to specific signals, we speculated that DEV pUL47 as a physiological phosphorylation substrate of cellular or viral protein kinase to mediate its nuclear localization. Therefore, whether DEV pUL47 is modified by other protein kinase need further investigation.

The host innate immune responses are triggered by recognition of pathogen-associated molecular patterns (PAMPs) via host–pathogen recognition receptors (PRRs), leading to induction of IFNs and pro-inflammatory cytokines. IFNs then activate the Janus kinase-signal transducer and activator of transcription (JAK-STAT) signalling pathway, resulting in transcription of numerous IFN-stimulated genes (ISGs). However, viruses can establish an infection in the host cells by overcoming the various innate defence mechanisms [65]. HSV-2 ICP27 directly associates with IRF3 [66], and US11 targets TBK1 to evade the antiviral innate immune response [67]. DEV is immunosuppressive and persistently infects monocytes/macrophages [68], but the mechanism of immune evasion by DEV is unknown. In our study, we first found that DEV pUL47 significantly inhibits the IFN-β signalling induced by poly(I:C) and downregulates the STAT1-induced ISG expression. Moreover, we also found that DEV pUL47 interacts with STAT1, similar to VP8 [11], but the detailed mechanism requires further exploration. Our data provide new insights into viral protein antagonizing host innate immune response in DEV infection.

In conclusion, the DEV UL47 gene is a late gene encoding an abundant structural protein assembled in virion. DEV pUL47 is mainly distributed in the nucleus of infected cells and contains two NLS motifs (residues 40 to 50 and 768 to 777). Moreover, DEV pUL47 inhibits IFN-β signalling by targeting STAT1 in the cytoplasm.

## Data Availability

The datasets analysed in this study are available from the corresponding author upon reasonable request.

## Abbreviations

β-Gal: beta-galactosidase
BoHV-1: bovine herpesvirus 1
DEV: duck enteritis virus
DP: duck plague
EGFP: enhanced green fluorescent protein
emPAI: exponentially modified protein abundance index
HSP60: heat shock protein-60
HSV-1: herpes simplex virus-1
ILTV: infectious laryngotracheitis virus
IFN: interferon
ISG: interferon-stimulated gene
MDV: Marek’s disease virus
NanoBiT: nanoLuc Binary Technology
NLS: nuclear localization sequence
NPCs: nuclear pore complexes
OASL: oligoadenylate synthetase-like
PAMPs: pathogen-associated molecular patterns
PRRs: pathogen recognition receptors
poly(I:C): polyriboinosinic:polyribocytidylic acid
PRV: pseudorabies virus
RT-qPCR: real-time quantitative reverse-transcription PCR
STAT1: signal transducer and activator of transcription-1
VZV: varicella-zoster virus
